# Targeted Whole Genome Sequencing of African Swine Fever Virus and Classical Swine Fever Virus on the MinION Portable Sequencing Platform

**DOI:** 10.3390/pathogens14080804

**Published:** 2025-08-13

**Authors:** Chester D. McDowell, Taeyong Kwon, Patricia Assato, Emily Mantlo, Jessie D. Trujillo, Natasha N. Gaudreault, Leonardo C. Caserta, Igor Morozov, Jayme A. Souza-Neto, Roman M. Pogranichniy, Diego G. Diel, Juergen A. Richt

**Affiliations:** 1 Department of Diagnostic Medicine/Pathobiology, College of Veterinary Medicine, Kansas State University, Manhattan, KS 66506, USA; 2 Department of Population Medicine and Diagnostic Sciences, College of Veterinary Medicine, Cornell University, Ithaca, NY 14853, USA; 3 Veterinary Diagnostic Laboratory, College of Veterinary Medicine, Kansas State University, Manhattan, KS 66506, USA

**Keywords:** African swine fever, classical swine fever, next-generation sequencing, whole genome

## Abstract

African swine fever virus (ASFV) and classical swine fever virus (CSFV) are important transboundary animal diseases (TADs) affecting swine. ASFV is a large DNA virus with a genome size of 170–190+ kilobases (kB) belonging to the family *Asfarviridae*, genus Asfivirus. CSFV is a single-stranded RNA virus with a genome size of approximately 12 kB, belonging to the family *Flaviviridae*, genus Pestivirus. Outbreaks involving either one of these viruses result in similar disease syndromes and significant economic impacts from: (i) high morbidity and mortality events; (ii) control measures which include culling and quarantine; and (iii) export restrictions of swine and pork products. Current detection methods during an outbreak provide minimal genetic information on the circulating virus strains/genotypes that are important for tracing and vaccine considerations. The increasing availability and reduced cost of next-generation sequencing (NGS) allow for the establishment of NGS protocols for the rapid identification and complete genetic characterization of outbreak strains during an investigation. NGS data provides a better understanding of viral spread and evolution, facilitating the development of novel and effective control measures. In this study, panels of primers spanning the genomes of ASFV and CSFV were independently developed to generate approximately 10 kB and 6 kB amplicons, respectively. The primer panels consisted of 19 primer pairs for ASFV and 2 primer pairs for CSFV, providing whole genome amplification of each pathogen. These primer pools were further optimized for batch pooling and thermocycling conditions, resulting in a total of 5 primer pools/reactions used for ASFV and 2 primer pairs/reactions for CSFV. The ASFV primer panel was tested on viral DNA extracted from blood collected from pigs experimentally infected with ASFV genotype I and genotype II viruses. The CSFV primer panel was tested on 11 different strains of CSFV representing the three known CSFV genotypes, and 21 clinical samples collected from pigs experimentally infected with two different genotype 1 CSF viruses. ASFV and CSFV amplicons from optimized PCR were subsequently sequenced on the Oxford Nanopore MinION platform. The targeted protocols for these viruses resulted in an average coverage greater than 1,000X for ASFV, with 99% of the genome covered, and 10,000X–20,000X for CSFV, with 97% to 99% of the genomes covered. The ASFV targeted whole genome sequencing protocol has been optimized for genotype II ASF viruses that have been responsible for the more recent outbreaks outside of Africa. The CSFV targeted whole genome sequencing protocol has universal applications for the detection of all CSFV genotypes. Protocols developed and evaluated here will be essential complementary tools for early pathogen detection and differentiation, as well as genetic characterization of these high-consequence swine viruses, globally and within the United States, should an outbreak occur.

## 1. Introduction

Transboundary animal diseases (TADs) are of great concern to the global agricultural systems. TADs can easily traverse national and international borders, resulting in devastating economic impacts due to high morbidity and mortality events, costly control strategies, and imposed trade restrictions. This study focuses on two TADs that have significant impacts on the global pork industry. One of these TADs currently in the spotlight is African swine fever virus (ASFV), which is the causative agent of African swine fever, a contagious viral disease of domestic swine and wild boar (Sus Scrofa) that can cause high mortality in affected swine populations. ASFV belongs to the *Asfarviridae* family, genus Asfivirus [1,2]. The virus particle of ASFV encapsulates a 170–192 kB double-stranded DNA genome encoding more than 150 genes [3,4,5,6]. Historically, there are 24 genotypes of ASFV with varying virulence between and within genotypes; recent retrospective studies of ASFV sequences suggest that there are only 6 to 7 ASFV genotypes, including the genotype I/II virus recombinant virus isolated recently in China [7,8]. Since the 2007 outbreak in Georgia, ASFV’s global presence has expanded to central and eastern Europe, Asia, and the Caribbean [9]. Another important TAD is classical swine fever virus (CSFV), the causative agent of classical swine fever or historically termed “hog cholera” [10]. CSFV belongs to the *Flaviviridae* family, genus Pestivirus; the enveloped virus particle contains a non-segmented single-stranded positive-sense RNA genome of approximately 12 kB encoding 13 viral proteins [11,12]. Currently, there are three known genotypes with over 13 sub-genotypes [13]. CSFV was first described in the United States and has since been eradicated from the U.S. swine population. However, CSFV remains endemic in many countries around the world. The risk of introduction and reintroduction into currently CSFV-free countries remains high. Until these two TADs are controlled and eventually eradicated, they will continue to threaten the global pork industry. Currently, rapid detection and implementation of strict mitigation strategies are the most effective way of impeding global spread.

Currently, the World Organization for Animal Health (WOAH) recommends the use of qPCR or RT-qPCR for the detection of ASFV and CSFV, respectively [14,15]. These methods offer high specificity and sensitivity with shorter turnaround times compared to traditional diagnostic techniques such as antigen ELISA and virus isolation. However, these techniques are unable to provide genotypic information of circulating viral strains in an outbreak. Traditionally, this information is obtained by sequencing specific genes or regions that differ between genotypes. In the case of ASFV, genotyping is based on the sequencing of the C-terminal variable region of the B646L gene that encodes the major capsid protein, p72 (478 bp) [16,17]. ASFV strains are further characterized by sequencing the E183L (inner membrane protein p54), the central hypervariable region (CVR) of B602L (viral chaperone), and the EP402R (CD2v) and EP153R (C-type lectin) genes [18,19]. Genotyping CSFV is achieved by sequencing the 5’-NTRs, the partial or full E2 gene, the partial NS5B gene, or the whole viral genome [15,20]. Due to the size of the ASFV genome and the high cost of sequencing in previous years, partial sequencing of these two agents was sufficient for genetically characterizing circulating or outbreak strains. However, partial sequencing provided only limited genetic information, has the potential for erroneous characterization of viral strains, and limits researchers’ ability to fully understand the viral evolution of these pathogens [7]. Recent studies by Forth et al. investigating the genetic landscape of ASFV strains circulating in Germany found that there are multiple variants that have diverged from the original outbreak strain [21], supporting the critical need for whole genome analyses even for ASFV. 

The rapid advances in sequencing technologies have greatly reduced the cost and time needed to obtain whole genome sequences of these important pathogens and other viral agents. The development of the Oxford Nanopore MinION, a portable sequencing platform, has drastically changed the landscape of sequencing. The MinION platform uses a pore-based sequencing technology that has eliminated read length restrictions observed with previous sequencing technologies. This advancement has allowed for the development of targeted long-read amplicon-based sequencing protocols. The MinION platform is relatively inexpensive compared to the bench-top sequencing platforms, increasing the availability of sequencing technology [22]. 

In this study we aimed to develop targeted whole genome sequencing protocols using the MinION sequencing platform for the complete genetic characterization of ASFV and CSFV. The protocols are designed to rapidly obtain whole genome sequences of these two viruses from clinical samples within 36 to 48 h. They will aid in increasing the availability of whole genome sequences of ASFV and CSFV and will support efforts to provide additional genetic information to better understand viral evolution and aid in the development of novel diagnostics and effective vaccines. 

## 2. Materials and Methods

### 2.1. Sample Ethics Statement

All experiments involving African swine fever virus (ASFV) and classical swine fever virus (CSFV) were conducted in the BSL-3 laboratory at the Biosecurity Research Institute (BRI) at Kansas State University (KSU) in Manhattan, KS. All animal studies and experiments were approved and performed under the KSU Institutional Biosafety Committee (IBC, Protocol #s: 1651, 1668, and 1787, 12 November 2024) and the Institutional Animal Care and Use Committee (IACUC, Protocol #s: 4265 and 4363, 21 February 2020) in compliance with the Animal Welfare Act. The ASFV and CSFV clinical samples used in this study were acquired from experimentally infected pigs at KSU-BRI and cell culture-derived virus stocks (Tables 3–5). 

### 2.2. Nucleic Acid Extraction and Quantitative Real-Time PCR

#### 2.2.1. African Swine Fever Virus DNA Extraction and Quantitation

ASFV-specific DNA was detected and quantified in EDTA blood and spleens from experimentally infected pigs with ASFV strains Armenia/2007, Mongolia/2019 [23], or E70 by quantitative real-time PCR (qPCR) assay as previously described [23,24]. ASFV DNA was extracted using two different automated extraction systems, one for EDTA blood and one for spleen. For EDTA blood, total DNA was extracted using the MagMax Pathogen RNA/DNA kit (ThermoFisher Scientific, Waltham, MA, USA) using the Kingfisher^TM^ Duo Prime purification system, an automated magnetic bead extraction system, following the manufacturer’s protocol with minor modifications. Briefly, EDTA blood was mixed 1:1 with Buffer AL (Qiagen; Carlsbad, CA, USA) and heated at 70 °C for 10 min. Subsequently, 200 µL was mixed with 20 µL of bead mix consisting of an equal volume of nucleic acid binding beads and lysis enhancer. The mixture was gently mixed by pipetting and incubated for 2 min at room temperature. Phosphate-buffered saline (PBS, 100 µL) was added to the tube, and the entire volume was added to the extraction plate well containing 400 µL lysis buffer, binding buffer, and isopropyl alcohol. DNA-bound beads were washed twice with 300 µL of wash solution 1, once with 450 µL of wash solution 2, and once more with 450 µL of 200-proof molecular grade ethanol. The DNA-bound beads were dried for 5 min prior to being eluted in 100 µL elution buffer. Negative (molecular grade water/PBS) and positive (ASFV p72 plasmid) controls were included with each extraction.

The spleen samples were processed into 20% w/v tissue homogenates in Buffer ATL (Qiagen; Carlsbad, CA, USA) with a subsequent 30 min Proteinase K (80 µg, Qiagen; Carlsbad, CA, USA) digestion at 56 °C prior to nucleic acid extraction. Total DNA was extracted from tissue homogenates using the GeneReach Taco Mini Automatic Nucleic Acid Extraction System (GeneReach Biotechnology Corp., Taichung, Taiwan). Briefly, tissue homogenates were mixed at a 1:1 ratio with Buffer AL. Then, 200 µL of tissue homogenate and Buffer AL mixture were added to the extraction plate wells containing 50 µL of magnetic, 500 µL lysis buffer, 100 µL phosphate-buffered saline (PBS), followed by the addition of 200 µL isopropyl alcohol to each well. DNA-bound beads were washed twice with 750 µL of wash buffer A, once with 750 µL wash buffer B, and once more with 750 µL of 200-proof molecular grade ethanol. The DNA-bound beads were dried for 5 min prior to being eluted in 100 µL elution buffer. Negative (molecular grade water/PBS) and positive (ASFV p72 plasmid) controls were included with each extraction.

The ASFV DNA extracted from EDTA blood and spleens was detected and quantified using the primers and probe for the detection of the p72 ASFV gene as previously described by Zsak et al. [25], using a validated protocol performed with the PerfeCTa^®^ FastMix^®^ II (Quanta Biosciences, Gaithersburg, MD, USA) on the CFX96 Touch^TM^ Real-Time PCR Detection System (Bio-Rad, Hercules, CA, USA) as previously described [24]. Duplicate qPCR reactions were performed. Each PCR run included negative and positive controls, which consisted of nuclease-free molecular grade water and ASFV positive amplification control (p72 plasmid), respectively. 

#### 2.2.2. Classical Swine Fever Virus RNA Extraction and Quantitation

Specific CSFV RNA was detected and quantified from the serum, tonsils, and a mandibular lymph node of pigs experimentally infected with either Brescia or Bavaro strains of CSFV and from cell culture supernatant of 11 CSFV viral stocks by quantitative real-time reverse transcriptase PCR (RT-qPCR) described by Eberling et al. [26]. Prior to nucleic acid extraction, tissue homogenates were prepared from the tonsils and a mandibular lymph node using the same method as for ASFV spleens described above. Viral RNA was extracted from cell culture supernatant, serum, and tissue homogenates using the GeneReach Taco Mini Automatic Nucleic Acid Extraction System (GeneReach Biotechnology Corp., Taichung, Taiwan) as described above for the ASFV spleen samples, except with Buffer AL substituted by Buffer RLT (Qiagen; Carlsbad, CA, USA). Negative (molecular grade water/PBS) and CSFV-positive extraction controls (CSF phagemid provided by USDA-APHIS FADDL) were included with each extraction.

The CSFV RNA extracted from cell supernatant, serum, mandibular lymph node, and tonsils was detected and quantified using the primers and probe for detection of CSFV described by Eberling et al. using a validated protocol performed with qScript XLT 1-step RT-qPCR ToughMix (Quanta Biosciences, Gaithersburg, MD, USA) on the CFX96 Touch^TM^ Real-Time PCR Detection System (Bio-Rad, Hercules, CA, USA). Briefly, the final reaction mixture consisted of 2.5 µL of viral RNA combined with 17.5 µL master mix, including 0.2 µM forward primer, 0.4 µM reverse primer, and 0.2 µM probe. The thermocycling conditions were as follows: 50 °C for 20 min, 95 °C for 5 min, followed by 45 cycles at 95 °C for 10 s and 60 °C 1 min. Duplicate qPCR reactions were performed. Each PCR run included negative and positive controls, which consisted of nuclease-free molecular grade water and CSFV positive amplification control (CSF phagemid supplied by USDA-APHIS FADDL), respectively.

### 2.3. Primer Design

#### 2.3.1. African Swine Fever Virus

**Figure 1:**
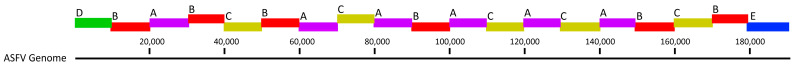
Genome map of the ASFV genome displaying the nineteen 10 kB amplicons. The colored bars represent the 5 primer pools: A: purple, B: red, C: yellow, D: green, E: blue.

A panel of 19 ASFV primer pairs (Table 1 and Figure 1) was designed based on the ASFV genotype II Georgia 2007 genome using Primer-BLAST 2.13.0 (NCBI), Snapgene (GSL Biotech LLC, Boston, MA, USA), and CLC Genomic Workbench 23.0.1 (Qiagen; Carlsbad, CA, USA). The tiled primers were designed to generate approximately 10 kB amplicons with 150–200 base pairs (bp) overlaps at the 3’ and 5’ ends. All primers were designed with similar melting temperatures, allowing for the use of one set of thermocycling conditions for all 19 reactions and primer pooling applications. 

#### 2.3.2. Classical Swine Fever Virus

**Figure 2:**
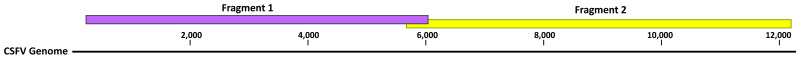
Genome map of CSFV genome displaying the two approximately 6 kB amplicons generated from the two primer pairs.

A panel of 2 CSFV primer pairs (Table 2) were designed using CLC genomics workbench 23.0.1 (Qiagen; Carlsbad, CA, USA) to generate approximately 6 kB fragments with a 380 bp overlap (Figure 2). The primer design module within the CLC Genomic Workbench 23.0.1 (Qiagen; Carlsbad, CA, USA) was used to identify candidate primers. The candidate primer sequences were compared to an alignment of multiple CSFV genome sequences to confirm binding sites were in conserved or low diversity regions within the CSFV genome. Degenerate nucleotides were added to primer sequences in regions of diversity for amplification of different CSFV genotypes. All primers were designed to have similar melting temperatures, so thermocycling conditions were consistent for the two primer pairs.

### 2.4. Whole Genome Amplification

#### 2.4.1. African Swine Fever Virus 

PCR reactions for all 19 primer pairs were tested individually for specificity and optimization of thermocycling conditions using ASFV viral DNA isolated from experimentally infected pig samples described above. Individual primer sets tested in 20 µL PCR reactions consisted of 10 µL Platinum Superfi II Green PCR MasterMix (ThermoFisher Scientific, Waltham, MA, USA), 2 µL of 20 µM primer pools (final primer concentration of 2 µM), 2 µL of viral DNA, and molecular grade water. The following thermocycling conditions were used: 98 °C for 30 s, followed by 35 cycles at 98 °C for 10 s, 60 °C for 10 s, 68 °C for 5 min, followed by a final extension step of 72 °C for 5 min. Correct amplicon sizes were confirmed by gel electrophoresis. Once optimal thermocycling conditions were achieved, 5 primer pools (pools A–E) were optimized to reduce the number of reactions needed for whole genome amplification (Figure 1); primer sequences and associated primer pools are provided in Table 1. Working stocks of primer pools contained 20 µM of each primer. ASFV amplicons were generated using the 5 primer pools using the following 40 µL PCR reactions consisting of 20 µL Platinum Superfi II Green PCR MasterMix (ThermoFisher Scientific, Waltham, MA, USA), 4 µL 20 µM primer pools (final primer concentration of 2 µM), 4 µL of viral DNA, and molecular grade water. The following thermocycling conditions were used: 98 °C for 30 s, followed by 35 cycles at 98 °C for 10 s, 60 °C for 10 s, 68 °C for 5 min, followed by a final extension step of 72 °C for 5 min. Amplicon pools were individually purified using Beckman Coulter AMPure XP beads (Beckman Coulter, Indianapolis, IN, USA) following the manufacturer’s protocol. Following the PCR clean-up with AMPure beads, the DNA concentration for each amplicon pool was quantified using the Invitrogen Qubit 4 Fluorometer (ThermoFisher Scientific, Waltham, MA, USA) with the broad-range dsDNA quantification kit. The five amplicon pools A–E were combined into one tube at a 1:1:1:1:1 ratio. The final pool was used for sequencing library preparation.

#### 2.4.2. Classical Swine Fever Virus 

A two-step RT-PCR was used to amplify the two 6 kB amplicons. Firstly, viral cDNA was generated for each fragment individually using the LunaScript RT Master Mix kit (primer-free) (New England Biolabs, Ipswich, MA, USA) using a 20 µL reaction consisting of 4 µL of LunaScript RT Master Mix, 1 µL of CSFV reverse primer (10 µM), 5 µL of viral RNA, and molecular grade water. The reaction was completed using the following thermocycling conditions: 55 °C for 10 min followed by 95 °C for 1 min. Following cDNA synthesis 5 µL of viral cDNA was added to a 45 µL PCR master mix consisting of 25 µL of Q5 High-Fidelity 2X Master Mix (New England Biolabs, Ipswich, MA, USA), 5 µL of 10 µM primer pools (final primer concentration of 1 µM), and molecular grade water. The reaction was completed using the following thermocycling conditions: 98 °C for 1 min, followed by 35 cycles at 98 °C for 10 s, 60 °C for 30 s, 72 °C for 5 min, followed by a final extension step of 72 °C for 5 min and hold at 4 °C. Amplicon size was confirmed by gel electrophoresis. Amplicons were individually purified using Beckman Coulter AMPure XP beads (Beckman Coulter, Indianapolis, IN, USA) following the manufacturer’s protocol. Following the PCR clean-up with AMPure beads, the DNA concentration for each amplicon was quantified using the Invitrogen Qubit 4 Fluorometer (ThermoFisher Scientific, Waltham, MA, USA) with the broad-range dsDNA quantification kit. The two amplicons were combined into one tube at a 1:1 ratio. The final pool was used for sequencing library preparation.

### 2.5. Sequencing Library Preparation and MinION Sequencing

Sequencing libraries for both ASFV and CSFV were prepared using the Oxford Nanopore Rapid Barcoding Kit 96 (Oxford Nanopore Technologies, Oxford, UK) following the manufacturer’s protocol. For ASFV, sequencing libraries consisting of 6 barcoded samples were prepared. For CSFV, sequencing libraries consisting of up to 24 barcoded samples were prepared. Barcoded libraries were loaded onto the Oxford Nanopore MinION R9 flowcell (Oxford Nanopore Technologies, Oxford, UK) following manufacturer’s protocol. The MinION was run for 16 h with a read length inclusion of greater than 1 kB. Following completion of the run, base calling and demultiplexing were completed using the Oxford Nanopore MinKNOW software (https://nanoporetech.com/document/experiment-companion-minknow, accessed on 14 July 2025) (Oxford Nanopore Technologies, Oxford, UK). Parsed reads were imported into CLC Genomics Workbench 23.0.1 (Qiagen; Carlsbad, CA, USA). ASFV and CSFV sequencing reads were mapped to their respective reference genomes for coverage graph generation and mapping statistics. For several CSFV strains, de novo assembly was completed, and contigs were aligned using BLAST to determine the most similar available CSFV genome, which served as the reference sequence for the analysis described above. CSFV genome sequences were selected based on a BLAST search with a 95% percent identity threshold as well as matching genotype and subgenotypes.

## 3. Results

### 3.1. ASFV AmpliSeq Protocol

**Figure 3:**
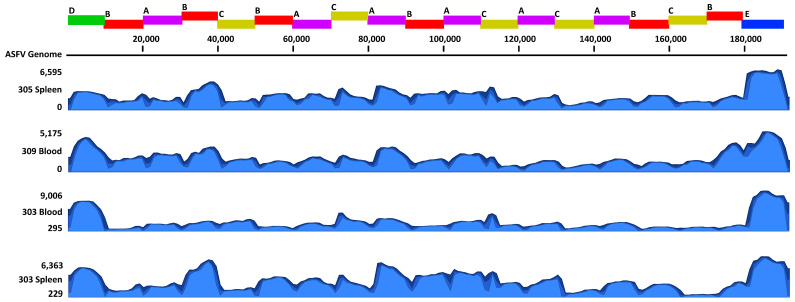
Coverage graphs of reads mapped to the ASFV genome. The amplicon schematic displayed on top is aligned with the coverage graphs below, indicating the 19 amplicons and associated pools (A–E). The graphs shown are a subset of samples tested using the ASFV AmpliSeq protocol. These samples include a blood and spleen sample from a pig experimentally infected with ASFV Armenia/2007 (#305 and #309) or ASFV Mongolia/2019 (#303).

The ASFV Ampliseq protocol was tested on 20 different ASFV samples. The samples included 19 clinical samples collected from pigs experimentally infected with ASFV genotype I E70 virus, ASFV genotype II Armenia/2007 and Mongolia/2019 viruses, as well as Armenia/2007 propagated in cell culture (see Table 3). The selected ASFV samples had Ct values for the ASFV p72 gene ranging from 14 to 33; samples with a Ct of less than 25 were diluted 1:10 to prevent PCR inhibition. Sequencing reads for genotype II samples were mapped to their respective ASFV genomes, Armenia/2007 or Mongolia/2019 (Figure 3). Sequencing reads for the ASFV genotype I E70 sample were mapped to a similar genotype I virus, E75. The average coverage of read mapping was greater than 1,000X, covering 99% of the ASFV genome except for samples from animals #304, #190, and #79 (Table 3 and Supplementary Table S1). Animal #304 had a high Ct value, which may have contributed to the drop in coverage. Furthermore, the DNA samples may have been degraded from the freeze/thaws necessary to perform other assays. Overall, following the 16 h MinION runs with 6 samples per run, on average, 220,000 sequencing reads per sample were obtained with an average read length of 2.5 kB and 72% of reads mapping to the reference genome. For the genotype II viruses ASFV Armenia 2007 and Mongolia 2019, 99% of the reference genome was covered with an average coverage of 1,764X. For ASFV genotype I E70 strain, ~59,000 reads mapped to 85% of the genome were covered with an average coverage of 1,081X. The regions that were not amplified and sequenced ranged in size from 8,000 to 20,000 bp, with the largest region at the 5’ end, which is a region that encodes numerous multiple gene families (MGFs). These regions may not have been amplified due to sequence differences between genotype I and II viruses at the primer binding sites. The ASFV AmpliSeq protocol obtained the near full-length genome of genotype II viruses with 99% coverage and ~1,000X coverage for the tested samples. The ASFV AmpliSeq protocol will allow researchers to investigate the evolution of genotype II viruses in detail, which have resulted in the most recent ASFV global outbreaks. Importantly, this protocol will provide sufficient genetic information for identifying genotype I/II recombinant ASFV viruses, which have recently been identified.

### 3.2. CSFV AmpliSeq Protocol

**Figure 4:**
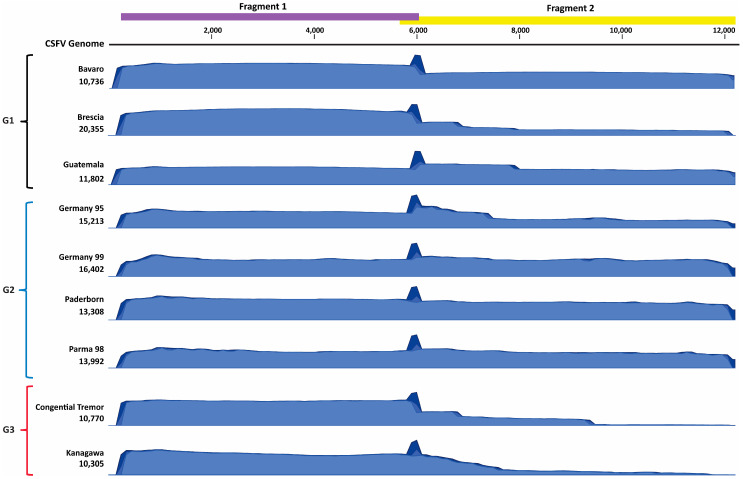
Coverage graphs for CSFV cell-culture derived viral stocks sequenced with CSFV AmpliSeq. The amplicon schematic displayed on top is aligned with the coverage graphs below, indicating the 2 amplicons. The graphs shown are a subset of viral stocks tested, which represent the 3 known genotypes of CSFV. Genotype 1 (G1): Bavaro, Brescia, Guatemala. Genotype 2 (G2): Germany 95, Germany 99, Paderborn, and Parma 98. Genotype 3 (G3): Congenital Tremor and Kanagawa.

Two primer sets were designed to amplify approximately 6 kB fragments of all known CSFV genotypes. The primers were tested against 5 CSFV strains isolated from cell culture, which included genotype 1 (Brescia, Bavaro, Guatemala), genotype 2 (Germany 1999), and genotype 3 (Congenital Tremor strain) viruses for optimizing thermal cycling conditions and suitability to amplify viruses representing the three known genotypes. The two primer sets amplified the correct size amplicons for all the CSFV strains tested, which was confirmed by gel electrophoresis. Smaller amplicons were observed after RT-PCR for fragment 2, likely due to partial or non-specific amplification; these smaller amplicons were excluded during the sequencing run using appropriate built-in bioinformatics filters. The efficiency of amplification for genotype 3 viruses was reduced, as observed by decreased concentrations of fragment 2 following RT-PCR. Following sequencing of the amplicons using the two primer sets, the average coverage ranged from 10,770X to 20,356X (Figure 4, Table 4, and Supplementary Table S2). The large spike in coverage observed in the coverage graphs is a result of the overlapping segment between the two primer pairs. We observed a significant reduction in coverage for fragment 2 of the Congenital Tremor strain of CSFV, with the depth of coverage reduced to 500–1,000 (Figure 4 and Table 4), most likely related to primer mismatches and/or higher sample Ct value. The CSFV AmpliSeq protocol was also tested on six additional cell-derived CSFV stocks: Alfort, PAV250, Paderborn, Parma98, Germany 1995, and Kanagawa (Table 4). For the 11 strains tested, there was an average coverage of 12,278X with an average read length of 1.6 kB covering 97–99% of the genome (Table 4). Similarly to the Congenital Tremor strain, the other genotype 3 strain, Kanagawa, also had reduced amplification of fragment 2 with a reduction in coverage depth to 2,000X. The reduced depth of coverage of fragment 2 for both genotype 3 strains is most likely related to primer mismatches and/or higher Ct values. Despite the reduction in depth of coverage for fragment 2, the depth is sufficient for obtaining the near full-length genomes of genotype 3 viruses.

To evaluate the use of this protocol for field application, we tested the protocol on clinical samples collected from pigs experimentally infected with two different genotype 1 CSFV strains, Bavaro and Brescia (see Table 5 and Supplementary Table S3). The Ct values for these samples ranged from 16 to 29. The clinical samples included serum collected on 5 and 7 DPC, tonsils collected at 11 DPC, and one mandibular lymph node collected on 5 DPC. The average coverage for the clinical samples was 15,496X with an average read length of 1.7 kB, covering 98–99% of the genome. The coverage of the clinical samples was similar to the cell culture-derived virus stocks. The CSFV AmpliSeq protocol can be used to obtain near full-length CSFV genomes from clinical samples and cell-derived virus stocks representing the three currently known CSFV genotypes. While a reduction in coverage of fragment 2 was observed for genotype 3 viruses and samples with Ct values greater than 30, the depth of coverage was sufficient for phylogenetic characterization.

## 4. Discussion

Historically, ASFV and CSFV were genetically characterized and phylogenetically grouped based on partial genetic sequences due to limited sequencing capabilities. For ASFV, the community relied heavily on the partial sequencing of the major capsid protein (p72) for genotyping [16,17]. Sequencing of p54, the variable region of B602L, and CD2v and C-type lectin [18,27], combined with p72 allows for further characterization of ASFV strains. These methods provide vital information for identification and outbreak investigations, but only provide limited genetic information on this large viral genome. Recent advances in sequencing technologies have allowed researchers to obtain full genome sequences of ASFV through direct sequencing approaches [28]. This is a resource-intensive method resulting in low coverage of the viral genome due to the overwhelming presence and sequencing of the host genome; this limitation was observed in our preliminary approach, where direct sequencing of ASFV resulted in a decreased number of mapped reads and lower depths of coverage. Due to the large genome of ASFV, amplicon-based sequencing has been difficult due to the need for numerous PCR reactions. The protocol developed in this study uses an amplicon-based targeted sequencing method. We reduced the number of reactions needed to amplify the ASFV genome by generating long amplicons of approximately 10 kB; these reactions were further reduced by generating primer pools allowing for the amplification of multiple 10 kB amplicons in one reaction. The resulting protocol allows for whole genome amplification of ASFV in 5 reactions in 4 to 4.5 h. Amplicon pools were then sequenced with the Oxford Nanopore MinION platform. With this protocol, we obtained full-length genotype II ASFV genomes in 36–48 h with an average coverage of 1,000X at a cost of approximately 70 USD per sample. The protocol was also tested on a genotype I ASFV and resulted in sequences with approximately 85% of the genome covered. The lack of coverage for the genotype I virus was observed at the 5’ end (~40,000 bp) of the genome, which encodes MGFs. This could be overcome by developing universal or degenerative primers for these regions. We concluded that the ASF AmpliSeq protocol will allow researchers to assemble near full-length genome sequences of this large DNA virus to study viral evolution and perform detailed phylogenetic analyses.

Genetic characterization and phylogenetic analysis of CSFV historically relied on partial sequencing of the E2 gene, the 5’-NTR, or the NS5 gene [15,20]. However, with advances in sequencing technology and continuing reduction in cost for sequencing, the WOAH recommends full-length sequencing of the E2 gene or whole genome sequencing. Protocols are available for amplification of the full-length E2 gene and whole CSFV genome that can be applied for sequencing [29,30]. The protocol described by Leifer et al. for amplification of the whole CSFV genome requires multiple RT-PCR reactions due to the average amplicon size being ~1 kB. The protocol described in this study generates two ~6 kB amplicons, which serve to amplify the whole CSFV genome in two RT-PCR reactions. After amplification, the pooled amplicons were sequenced on the Oxford Nanopore MinION platform. With this protocol, we obtained the near full-length genomes of viruses representing three currently known CSFV genotypes isolated from cell culture with the average coverage greater than 10,000X in 36–48 h at a cost of approximately 30 USD per sample. The protocol was tested on clinical samples from pigs experimentally infected with two different genotype 1 viruses. The CSFV AmpliSeq protocol will reduce the cost and amount of sample required to obtain the whole genome sequences of CSFV, allowing for a more in-depth understanding of the genetic evolution of CSFV while providing the necessary genetic information required for phylogenetic characterization of CSFV.

This study utilized low Ct ASFV and CSFV samples for the development and optimization of both protocols. These protocols performed well with samples that have a Ct value < 30. Since viral titers can vary across clinical field samples, further investigation is needed to determine the performance of these protocols with samples from the field and samples with low virus loads. The other concern with clinical samples is potential primer cross-reactivity with host DNA. This effect is minimized since these protocols utilize virus-specific primers and downstream bioinformatic pipelines that would filter out any host genome sequences. The described protocols perform well with experimental clinical samples, but further investigation is needed to determine performance with clinical field samples.

## 5. Conclusions

The targeted whole genome sequencing protocols for ASFV and CSFV described in this study will provide vital tools for diagnostics and research of these two high-consequence swine pathogens that have far-reaching impacts on both the global swine industry and food security. These protocols will enable researchers to rapidly obtain the near full-length genomes of ASFV and CSFV, providing more complete genetic information for phylogenetic analysis and viral evolution studies of these pathogens. Further development is needed for the ASFV AmpliSeq protocol to obtain the complete genomes of all 24 known ASFV genotypes; however, the present protocol can detect ASFV genotypes I and II that have emerged and continue to spread outside of Africa. While this study focuses on utilizing the MinION sequencing platform, these protocols would also be compatible with other sequencing platforms such as PacBio and Illumina. The reduced cost and timeframe for these protocols will be important tools to assist in early pathogen detection and genetic characterization of these high-consequence swine viruses in outbreak and surveillance situations within the United States and globally.

## Figures and Tables

**Table 1:** ASFV primers and associated pools.

Primer	Sequence	Primer Pool	Position ^1^
ASFV-1-F	GCGTTCATTTCACAAGATGCG	D	378–398
ASFV-1-R	TGGGATTTGTACCAGCAGGG	D	10,532–10,551
ASFV-2-F	GGGCTTTGGGTCAGAACAGT	B	10,395–10,414
ASFV-2-R	ACAAGCTATACACGGCTCTCAG	B	20,921–20,942
ASFV-3-F	TGCATTCCGATATCTCATCATCTGT	A	20,802–20,826
ASFV-3-R	TACGGCGTTAGTGAAGGCTG	A	31,212–31,231
ASFV-4-F	CTTGGCACCTAGCTCTCGAC	B	31,090–31,109
ASFV-4-R	ACGATTTAGCACAACCATGCG	B	40,709–40,729
ASFV-5-F	GCCAATTCCTGAACCTGATGC	C	40,576–40,596
ASFV-5-R	AAACGTGTTGTCCCTCAGCA	C	50,671–50,690
ASFV-6-F	GAAACGACTGCACGAATGGG	B	50,546–50,565
ASFV-6-R	TGTCGTGCTTGTTTGGTACG	B	60,647–60,666
ASFV-7-F	TGAAACAATACCCCGGCCTC	A	60,513–60,532
ASFV-7-R	CATTGAACGCCCGCAACATC	A	70,924–70,943
ASFV-8-F	TTACCTCCTGCCGACCATTG	C	70,786–70,805
ASFV-8-R	TCCCTGTGTTTCTTGAGCGG	C	80,652–80,671
ASFV-9-F	AGACCAAACAGCAAAACGCA	A	80,520–80,539
ASFV-9-R	CAGTTGGTAGGGCTTGTGGT	A	90,711–90,730
ASFV-10-F	AGGATCCCTACAGCACGATG	B	90,583–90,602
ASFV-10-R	TTTTCCCGTCAAAGCAACCG	B	100,848–100,867
ASFV-11-F	GGGAAGCGGCACTGTACTAT	A	100,718–100,737
ASFV-11-R	AAGCAACTCAACCCGCAAAC	A	110,625–110,644
ASFV-12-F	GAAAGAAGGGTGTAGTCAAA	C	110,432–110,451
ASFV-12-R	ACACAAATGTCCAACTAGCTC	C	120,618–120,638
ASFV-13-F	GTCTCCTAGGGGCATAAGCG	A	120,460–120,479
ASFV-13-R	AGGCAGGGCTTAATGCAAGA	A	130,282–130,301
ASFV-14-F	GCGAGGAACAGAGCCCATAA	C	130,150–130,169
ASFV-14-R	AAAGCCTATTGACTGAACCCGT	C	140,875–140,896
ASFV-15-F	ATGAAAATACTCCCCGTGGCA	A	140,737–140,757
ASFV-15-R	GTATCAGCGGGGTAAGCAGT	A	150,238–150,257
ASFV-16-F	GCGAGCAAAGCTGAGATACG	B	150,100–150,119
ASFV-16-R	AACCTTTTCCGGGTACGTGA	B	160,703–160,722
ASFV-17-F	GCCGCTTTGCCTCATTTACG	C	160,565–160,584
ASFV-17-R	ACGTCCATCGTTCAAGGAGT	C	170,849–170,868
ASFV-18-F	ATTGCGTTGCGATCCAGTTC	B	170,712–170,731
ASFV-18-R	ACCCGAGAACGCTTCACAA	B	180,298–180,316
ASFV-19-F	AACAAGGGAACGATGGGAAG	E	179,039–179,058
ASFV-19-R	CTGCTGTAGGTGTCAAAGAT	E	190,225–190,244

^1^: Position is based on accession number FR682468.2.

**Table 2:** CSFV primers.

Primer	Sequence	Position ^1^
CSFV-1-F	AWGCCCAAGACRCACCTTAAYC	118–380
CSFV-1-R	GGTTGYGDCATCTGRCARAAGT	6,260–6,721
CSFV-2-F	ATAGGDGAGATGAAGGARGG	5,927–6,253
CSFV-2-R	TAGTGTGGTAACWYGAGGTAG	5,565–12,205

^1^: Position is based on accession number M31768.1.

**Table 3:** Sequencing data for ASFV samples tested with ASFV AmpliSeq.

Animal ID	DPC ^1^	Type	Strain	Genotype	Ct	Reference Length	Mapped Reads	Total Reads	% Reads Mapped	Average Coverage	% Reference Covered	Average Read Length
Arm07 Cos-1 ^2^	P3 ^3^	Cell culture	Arm07 ^4^	II	27	190,584	51,134	65,081	78.57	1,017.61	99	3,749
63	7	Blood	Arm07 ^4^	II	16	190,584	127,541	158,104	80.67	2,138.01	99	3,157
67	9	Blood	Arm07 ^4^	II	19	190,584	149,958	184,043	81.48	2,323.76	99	2,928
87	7	Blood	E70 ^6^	I	25	181,187	59,820	91,435	97.37	1,081.24	85	3,357
190	7	Blood	Arm07 ^4^	II	15	190,584	60,571	80,273	75.46	864	99	2,681
192	7	Blood	Arm07 ^4^	II	16	190,584	85,585	112,479	76.09	1,226.02	99	2,694
303	7	Blood	MNG-19 ^5^	II	19	187,868	120,141	166,122	72.32	2,124.92	99	3,306
303	7	Spleen	MNG-19 ^5^	II	19	187,868	155,498	207,421	74.97	2,511.41	99	3,020
304	7	Blood	Arm07 ^4^	II	25	190,584	64,340	308,436	20.86	440.05	96	1,242
305	7	Spleen	Arm07 ^4^	II	21	190,584	136,574	173,381	78.77	2,193.54	99	3,035
306	6	Blood	MNG-19 ^5^	II	22	187,868	423,361	575,567	73.56	2,313.04	99	1,019
307	3	Blood	MNG-19 ^5^	II	22	187,868	264,992	390,731	67.82	1,465.03	99	1,029
308	7	Blood	Arm07 ^4^	II	21	190,584	310,219	426,981	72.65	1,842.86	99	1,117
309	7	Blood	Arm07 ^4^	II	19	190,584	96,240	123,894	77.68	1,726.88	99	3,378
310	7	Blood	MNG-19 ^5^	II	19	187,868	66,069	88,936	74.29	1,079.36	99	3,047
311	7	Blood	Arm07 ^4^	II	19	190,584	98,755	129,042	76.53	1,676.69	99	3,187
312	7	Blood	Arm07 ^4^	II	20	190,584	131,299	173,421	75.71	2,299.50	99	3,297
314	7	Blood	MNG-19 ^5^	II	22	187,868	307,810	421,638	73	1,714.75	99	1,037
511	7	Blood	Arm07 ^4^	II	20	190,584	121,736	152,400	79.88	1,912.97	99	2,970
635	7	Blood	Arm07 ^4^	II	14	190,584	165,992	215,116	77.16	2,654.20	99	3,022

^1^ DPC: Days post challenge; ^2^ Arm07-Cos-1: ASFV Armenia 2007 passaged on Cos-1 cells; ^3^ P3: Passage 3; ^4^ Arm07: ASFV Armenia 2007, Accession Number: FR682468.2; ^5^ MNG-19: ASFV Mongolia 2019, Accession Number: OP467597.1; ^6^ E70: ASFV Espana 1970, Accession Number: FN557520.1.

**Table 4:** Sequencing data for 11 CSFV strains tested with CSFV AmpliSeq.

Strain	Genotype	Ct	Reference Length	Mapped Reads	Total Reads	% Reads Mapped	Average Coverage	% Reference Covered	Average Read Length
Alfort ^1^	1.1	33	12,298	85,869	128,711	66.7	8,643.29	97	1,235
Brescia ^2^	1.2	17	12,283	147,575	191,259	77.2	20,355.95	98	1,683
Bavaro ^3^	1.3	23	11,955	61,093	80,719	75.7	10,736.37	99	2,112
PAV250 ^2^	1.2	32	12,283	57,373	120,788	47.5	6,275.58	97	1,337
Guatemala ^3^	1.3	26	11,955	75,909	85,462	88.8	11,802.41	99	1,868
Paderborn ^4^	2.1	30	12,296	102,050	147,935	68.9	13,308.74	97	1,599
Parma 98 ^4^	2.2	31	12,296	128,430	173,185	74.2	13,992.47	97	1,333
Germany 1995 ^5^	2.3	32	12,296	112,426	151,928	73.9	15,213.59	97	1,659
Germany 1999 ^6^	2.3	29	12,296	123,679	142,929	86.5	16,402.40	97	1,627
Congenital Tremor ^7^	3.1	32	12,298	75,708	103,765	72.9	10,770.79	97	1,750
Kanagawa ^7^	3.4	34	12,298	87,635	146,523	59.8	10,305.39	97	1,447

Accession numbers of reference sequences: ^1^: NC_038912.1, ^2^: M31768.1, ^3^: MG655308.1, ^4^: GQ902941.1, ^5^: HM237795.1, ^6^: HQ148062.1, ^7^: KF669877.1.

**Table 5:** Sequencing data for clinical CSFV samples tested with CSFV AmpliSeq.

Animal ID	DPC ^1^	Sample Type	Strain	Genotype	Ct	Reference Length	Mapped Reads	Total Reads	% Reads Mapped	Average Coverage	% Reference Covered	Average Read Length
169	5	Serum	Brescia *	1.2	24.84	12,283	133,564	165,617	80.6	19,738.50	98	1,802
226	5	Serum	Brescia *	1.2	23.63	12,283	121,651	148,444	81.9	19,799.28	98	1,984
226	5	Mandibular LN ^2^	Brescia *	1.2	22.7	12,283	88,935	138,679	64.1	13,850.58	97	1,901
287	5	Serum	Brescia *	1.2	20.89	12,283	109,336	142,280	76.8	17,646.33	98	1,970
348	5	Serum	Bavaro ^‡^	1.3	28.83	11,955	98,559	106,690	92.4	15,503.91	99	1,888
353	5	Serum	Brescia *	1.2	20.09	12,283	107,481	133,827	80.3	15,333.07	98	1,739
354	5	Serum	Brescia *	1.2	21.26	12,283	112,049	143,418	78.1	15,997.95	98	1,742
358	5	Serum	Bavaro ^‡^	1.3	27.3	11,955	184,126	203,348	90.5	30,853.70	99	2,007
374	5	Serum	Bavaro ^‡^	1.3	29.15	11,955	88,296	106,370	83	11,465.05	99	1,563
3246	5	Serum	Bavaro ^‡^	1.3	26.43	11,955	118,471	131,482	90.1	18,171.38	99	1,841
3248	5	Serum	Bavaro ^‡^	1.3	29.36	11,955	89,295	100,880	88.5	10,791.20	99	1,445
169	7	Serum	Brescia *	1.2	16.1	12,283	75,350	94,675	79.6	10,040.89	98	1,625
287	7	Serum	Brescia *	1.2	16.9	12,283	132,993	167,506	79.4	17,255.71	98	1,582
348	7	Serum	Bavaro ^‡^	1.3	25.5	11,955	92,379	104,221	88.6	12,824.24	99	1,664
358	7	Serum	Bavaro ^‡^	1.3	26.1	11,955	88,799	103,837	85.5	13,585.76	99	1,833
374	7	Serum	Bavaro ^‡^	1.3	27.03	11,955	57,973	66,023	87.8	7,522.64	99	1,553
3246	7	Serum	Bavaro ^‡^	1.3	19.98	11,955	108,501	117,808	92.1	16,394.22	99	1,809
3248	7	Serum	Bavaro ^‡^	1.3	29.25	11,955	100,504	110,978	90.6	13,020.98	99	1,548
169	11	Tonsil	Brescia *	1.2	17	12,283	147,575	191,259	77.2	20,355.95	98	1,683
348	11	Tonsil	Bavaro ^‡^	1.3	23	11,955	61,093	80,719	75.7	10,736.37	99	2,112
3246	11	Tonsil	Bavaro ^‡^	1.3	25	11,955	88,948	103,779	85.8	14,534.34	99	1,955

^1^ DPC: Days post challenge, ^2^ LN: lymph node, accession numbers of reference sequences: *: M31768.1, ^‡^: MG655308.1.

## Data Availability

All the data is included within the manuscript.

## Supplementary Materials

The following supporting information can be downloaded at: https://www.mdpi.com/article/10.3390/pathogens14080804/s1, Table S1: Detailed Sequencing data for ASFV samples; Table S2: Detailed sequencing data for 11 CSFV strains; Table S3: Detailed sequencing data for clinical CSFV samples.
