# Supplementary material for: Targeted Whole Genome Sequencing of African Swine Fever Virus and Classical Swine Fever Virus on the MinION Portable Sequencing Platform
Source: Pathogens. 2025 Aug 13;14(8):804. doi: 10.3390/pathogens14080804 (PMC12389616; doi:10.3390/pathogens14080804)
Supplement: Supplementary file 1 [file pathogens-14-00804-s001.zip › pathogens-3790510-supplementary.pdf]

Table S1. Detailed Sequencing data for ASFV samples.

Animal ID	DPC[1]	Type	Strain	Average Coverage	Coverage Standard Deviation	Average Read Length	Read Length Standard Deviation
Arm07 Cos-1[2]	P3[3]	Cell culture	Arm07[4]	1017.61	637.20	3749	2567
63	7	Blood	Arm07[4]	2138.01	1504.40	3157	2136
67	9	Blood	Arm07[4]	2323.76	1187.07	2928	2122
87	7	Blood	E70[6]	1081.24	942.23	3357	2475
190	7	Blood	Arm07[4]	864	486.39	2681	2094
192	7	Blood	Arm07[4]	1226.02	642.36	2694	2103
303	7	Blood	MNG-19[5]	2124.92	1750.72	3306	2323
303	7	Spleen	MNG-19[5]	2511.41	1391.02	3020	2172
304	7	Blood	Arm07[4]	440.05	375.37	1242	1760
305	7	Spleen	Arm07[4]	2193.54	1197.64	3035	2207
306	6	Blood	MNG-19[5]	2313.04	1937.07	1019	1494
307	3	Blood	MNG-19[5]	1465.03	945.60	1029	1517
308	7	Blood	Arm07[4]	1842.86	1695.71	1117	1587
309	7	Blood	Arm07[4]	1726.88	992.14	3378	2315
310	7	Blood	MNG-19[5]	1079.36	703.55	3047	2142
311	7	Blood	Arm07[4]	1676.69	1549.94	3187	2282
312	7	Blood	Arm07[4]	2299.50	1901.52	3297	2329
314	7	Blood	MNG-19[5]	1714.75	1502.33	1037	1506
511	7	Blood	Arm07[4]	1912.97	1282.10	2970	2123
635	7	Blood	Arm07[4]	2654.20	1868.73	3022	2189

[1]DPC: Days post challenge; [2]Arm07-Cos-1: ASFV Armenia 2007 passaged on Cos-1 cells; [3]P3: Passage 3; [4]Arm07: ASFV Armenia 2007; [5]MNG-19: ASFV Mongolia 2019; [6]E70: ASFV E70.

Table S2. Detailed sequencing data for 11 CSFV strains.

Strain	Average Coverage	Coverage Standard Deviation	Average Read Length	Read Length Standard Deviation
Alfort	8643.29	8002.12	1235	1312
Brescia	20,355.95	4608.47	1683	1539
Bavaro	10,736.37	2681.90	2112	1651
PAV250	6275.58	4608.47	1337	1388
Guatemala	11,802.41	1972.77	1868	1630
Paderborn	13,308.74	2737.67	1599	1713
Parma 98	13,992.47	2881.76	1333	1568
Germany 1995	15,213.59	13,230.67	1659	1572
Germany 1999	16,402.40	7915.32	1627	1600
Congenital Tremor	10,770.79	7871.03	1750	1519
Kanagawa	10,305.39	7363.69	1447	1483

Table S3. Detailed sequencing data for clinical CSFV samples.

Animal ID	DPC[1]	Sample Type	Strain	Average Coverage	Coverage Standard Deviation	Average Read Length	Read Length Standard Deviation
169	5	Serum	Brescia	19,738.50	14,144.32	1802	1587
226	5	Serum	Brescia	19,799.28	12,713.63	1984	1640
226	5	Mandibular LN[2]	Brescia	13,850.58	13,712.31	1901	1615
287	5	Serum	Brescia	17,646.33	11,798.56	1970	1618
348	5	Serum	Bavaro	15,503.91	6050.56	1888	1616
353	5	Serum	Brescia	15,333.07	9432.26	1739	1566
354	5	Serum	Brescia	15,997.95	8961.11	1742	1583
358	5	Serum	Bavaro	30,853.70	4846.63	2007	1604
374	5	Serum	Bavaro	11,465.05	8877.05	1563	1537
3246	5	Serum	Bavaro	18,171.38	2995.35	1841	1681
3248	5	Serum	Bavaro	10,791.20	6058.30	1445	1453
169	7	Serum	Brescia	10,040.89	4274.32	1625	1588
287	7	Serum	Brescia	17,255.71	8875.12	1582	1552
348	7	Serum	Bavaro	12,824.24	2788.97	1664	1588
358	7	Serum	Bavaro	13,585.76	3945.07	1833	1597
374	7	Serum	Bavaro	7522.64	2520.70	1553	1459
3246	7	Serum	Bavaro	16,394.22	7915.33	1809	1581
3248	7	Serum	Bavaro	13,020.98	7669.82	1548	1391
169	11	Tonsil	Brescia	20,355.95	12,925.15	1683	1539
348	11	Tonsil	Bavaro	10,736.37	2681.90	2112	1652
3246	11	Tonsil	Bavaro	14,534.34	5407.39	1955	1654

[1]DPC: Days post challenge, [2]LN: Lymph Node.
